# Steady-State Clozapine and Norclozapine Pharmacokinetics in Maori and European Patients

**DOI:** 10.1016/j.ebiom.2017.11.030

**Published:** 2017-12-13

**Authors:** David B. Menkes, Paul Glue, Christopher Gale, Frederic Lam, Cheung-Tak Hung, Noelyn Hung

**Affiliations:** a Waikato Clinical Campus, Private Bag 3200, Hamilton, New Zealand; b University of Otago, PO Box 56, Dunedin 9054, New Zealand; c Zenith Technology, 156 Frederick Street, Dunedin 9016, New Zealand

**Keywords:** C_max_, maximum plasma concentration, AUC_0 − τ_, area under the curve from time 0 to the end of the dosing interval, t_max_, time to C_max_, C_min_, minimum plasma concentration, Clozapine, Norclozapine, Maori, Bioavailability, Pharmacokinetic, Metabolism

## Abstract

**Background:**

Clozapine is the most effective drug for treatment-resistant schizophrenia, but its use is limited by toxicity. Because ethnicity has been reported to affect clozapine metabolism, we compared its steady state pharmacokinetics in New Zealand Maori and European patients.

**Methods:**

Clozapine and norclozapine steady state bioavailability was assessed over 24 h under fasting and fed conditions in 12 Maori and 16 European patients treated for chronic psychotic illnesses with stable once-daily clozapine doses. Plasma clozapine and norclozapine concentrations were assessed using liquid chromatography with tandem mass spectrometry; pharmacokinetic parameters were calculated using standard non-compartmental methods, and compared using unpaired *t*-tests.

**Findings:**

Mean pharmacokinetic parameters (AUC, C_max_ and C_min_) for clozapine and norclozapine were virtually identical in Maori and European subjects, under both fed and fasted conditions.

**Discussion:**

Clozapine bioavailability does not vary between Maori and European patients, and thus does not need to be considered in prescribing decisions. Additional studies are needed to identify if there are differences between Maori and European populations for drugs metabolized by other enzyme pathways.

## Highlights

• Metabolism of clozapine, our most effective antipsychotic drug, was compared in New Zealand's two main ethnic groups.
• Clozapine pharmacokinetic parameters were similar in Maori and European patients.
• Accordingly, clozapine dosing does not need to take Maori or European ethnicity into account.

Clozapine is an essential medicine for treatment-resistant schizophrenia, but its use is limited by significant side-effects. We were interested to see if Maori and European patients in New Zealand differed in terms of clozapine pharmacokinetics, as this could influence both clinical response and side-effects. We found essentially no difference in clozapine pharmacokinetics between these groups, meaning that dosing decisions can be made based on clinical factors without regard to ethnicity.

## 1 Introduction

Compared with other antipsychotic drugs, clozapine is the most effective pharmacotherapy for schizophrenia; its use is generally restricted to treatment-resistant patients because of safety risks, notably agranulocytosis, myocarditis, and severe constipation (Barnes et al., 2011). Surveys have identified similar (Wheeler et al., 2008) or somewhat higher (Dey et al., 2016) rates of clozapine use in Maori compared to Europeans, the two dominant ethnic groups in New Zealand. Ethnicity has been shown to influence clozapine pharmacokinetics in some settings; for example, compared with Caucasians, lower daily clozapine doses are needed to achieve comparable blood levels in Asian (Ng et al., 2005) and Mexican patients (González-Esquivel et al., 2011). There are no published data on the pharmacokinetics of clozapine in Maori, although mean daily clozapine doses were similar in Maori and European patients in a retrospective cross-sectional survey (El-Badri and Mellsop, 2011). Effective treatment of schizophrenia in Maori is of particular importance given its apparently higher prevalence compared with the European population (Kake et al., 2008). This study used data from a bioequivalence trial of two clozapine formulations (Glue et al., 2012) in order to compare the pharmacokinetics of clozapine and its active metabolite norclozapine in Maori and European patients.

## 2 Materials and Methods

A detailed description of the bioequivalence study, including sample size calculation, is provided elsewhere (Glue et al., 2012). In brief, the study enrolled 30 male and female subjects, aged 18 to 55 years, established on stable doses of clozapine for at least 3 months. Subjects were required to have a body mass index (BMI) between 18 and 35 kg/m^2^, and be in good health. Clozapine was prescribed in multiples of 50 mg, taken as a single evening dose. Subjects provided written informed consent prior to participation. The study conformed to standards indicated by the Declaration of Helsinki; approval was provided by the New Zealand Multi-Region Ethics Committee (MEC/10/09/094). The study design involved an 11-day dosing period with one formulation, tablet or suspension, with pharmacokinetic blood sampling under fasting and fed conditions on days 10 and 11, respectively. Study subjects were then switched to the alternate formulation from days 12–22, with repeated pharmacokinetic sampling on days 21 and 22. The order in which formulations were administered was based on a computer-generated random code. The formulations were found to be bioequivalent under fasting and fed conditions (Glue et al., 2012). This report describes the pharmacokinetics of the tablet formulation (Clozaril®, Novartis) in Maori and European participants.

On Days 10 and 21, subjects were admitted to the study clinic at least 10 h prior to drug administration, and were discharged after the final blood draw in the morning of Days 12 and 23. Clozapine was administered after an 8-hour fast on Days 10 and 21, and was dosed under fed conditions on Days 11 and 22 after consumption of a standardized high-fat meal (Food and Drug Administration, 2002). All clozapine doses were administered with 240 mL water. There was no washout period between the two treatment periods. On Days 10, 11, 21 and 22, blood samples were collected at baseline and at 0.25, 0.5, 1, 1.5, 2, 2.5, 3, 3.5, 4, 5, 6, 8, 10, 12, 14, 16, 20 and 24 h after dosing. Following centrifugation, plasma samples were stored in polypropylene tubes at 70 °C until assayed using validated liquid chromatography with tandem mass spectrometry methods. Clozapine was assayed using a previously reported method (Glue et al., 2012). The norclozapine assay was similar although a different internal standard (*N*-desmethylmirtazapine) was used.

Pharmacokinetic parameters, calculated using standard non-compartmental methods, included maximum plasma concentration (C_max_), area under the plasma concentration–time curve from time 0 to the end of the dosing interval (AUC_0-τ_), time to C_max_ (t_max_), and minimum concentration (C_min_). Because patients were taking different daily doses of clozapine, all clozapine and norclozapine plasma concentrations and pharmacokinetic data were normalised to a dose of 100 mg.

Safety was monitored by assessing adverse events, laboratory test results, vital signs (heart rate and blood pressure), and electrocardiograms (ECGs).

Pharmacokinetic parameters were compared using unpaired *t*-tests or, as necessary, the Mann-Whitney *U* test, a non-parametric equivalent. Safety data were analysed using summary statistics. The sample size for the study was pragmatic. Post-hoc assessment of statistical power indicates that the study had at least 80% power to detect a 40% difference in AUC and a 30% difference in C_max_, based on fasted pharmacokinetic parameters.

## 3 Results

### 3.1 Subjects

Of 30 subjects in the original bioequivalence study, two of other ethnic origins were excluded from the present comparison of 16 European (13 male, 3 female) with 12 Maori participants (10 male, 2 female), see Table 1. Maori ethnicity was determined according to the New Zealand Census procedure (2001), and confirmed by participants identifying at least one (mean 2.9, range 1–4) Maori grandparent. Neither age nor BMI differed statistically between the two groups (European [mean ± standard deviation] age = 38.0 ± 9.0 years, BMI = 30.0 ± 5.0 kg/m^2^; Maori age = 37.6 ± 10.4 years, BMI = 32.2 ± 5.2 kg/m^2^). Among European participants there were 6 smokers and 10 nonsmokers, and in Maori, 8 smokers and 4 nonsmokers. The greater proportion of Maori than European smokers (67% vs. 37%) did not reach statistical significance (chi square = 1.31, p = 0.25).

**Table 1:** Demographic, prescription and smoking data.

Participant	Ethnicity	Gender	Age (years)	BMI (kg/m^2^)	Clozapine mg/day	Tobacco cigarettes/day	Concomitant medications
1	Maori	Male	42	24	400	20	Amitriptyline 25 mg twice daily
2	Maori	Male	46	32.5	100	20	Lamivudine 100 mg daily
							Pantoprazole 40 mg daily
3	Maori	Male	42	42	400	0^a^	Omeprazole 20 mg daily
							Metformin 850 mg daily
							Diclofenac 75 mg PRN
4	Maori	Male	28	38	350	16	Omeprazole 20 mg daily
							Sodium valproate 1.6 g daily
5	Maori	Male	30	31.4	450	15	Amisulpride 400 mg twice daily
6	Maori	Male	28	33.9	700	10	Venlafaxine 150 mg daily
7	Maori	Male	57	29.7	700	24	Lithium carbonate 1.5 g daily
8	Maori	Female	46	37	400	5	Quetiapine 50 mg daily
							Fluoxetine 20 mg
9	Maori	Male	27	33.4	400	0^a^	Nil
10	Maori	Male	48	29.8	400	0^a^	Risperidone 0.5 tab daily
							Benztropine 2 mg daily
11	Maori	Female	33	30.8	100	0^a^	Quetiapine 50 mg twice daily
							Loperamide 2 mg (if required)
							Propanolol 40 mg twice daily
12	Maori	Male	25	24	600	13	Nil
13	European	Male	42	22.8	400	0^a^	Citalopram 40 mg daily
							Simvastatin 10 mg daily
							Clonazepam 0.5 mg daily
14	European	Male	40	24.2	700	20	Clonazepam 2 mg daily
							Simvastatin 20 mg daily
15	European	Male	29	24	500	0^a^	Lithium carbonate 2.5 g daily
							Simvastatin 40 mg daily
16	European	Male	56	28	300	5	Omeprazole 20 mg daily
							Calcium carbonate 1.25 g daily
17	European	Male	41	29.7	400	8	Nil
18	European	Female	45	37.8	650	0^a^	Haloperidol 50 mg i.m. monthly
							Haloperidol 5 mg nocte PRN
							Cilazapril1.25 mg daily
							Pantoprazole 40 mg daily
19	European	Female	48	22.1	200	0^a^	Levonorgestrel 20 mcg daily
20	European	Male	48	37	600	20	Chlopromazine 100 mg daily
							Clonazepam 2 mg daily
							Levothyroxine 0.05 mg daily
							Sodium valproate 1.8 g daily
							Lithium carbonate 1.2 g daily
21	European	Male	29	28	350	0^a^	Nil
22	European	Male	40	35.4	600	0^a^	Sodium valproate 600 mg Nocte
							Amisulpiride 300 mg nocte
							Citalopram 40 mg daily
23	European	Male	38	32.8	100	0^a^	Omeprazole 20 mg daily
24	European	Male	29	27.7	350	0^a^	Omeprazole 40 mg daily
							Sodium valproate 1.4 g daily
							Simvastatin 20 mg daily
25	European	Male	26	32.4	400	0^a^	Nil
26	European	Female	39	32.5	350	0^a^	Metoprolol 95 mg daily
							Omeprazole 20 mg daily
							Atorvastatin 30 mg daily
27	European	Male	34	34.7	500	10	Amisulpride 600 mg twice daily
							Lithium carbonate 1.2 g daily
28	European	Male	24	32.2	500	5	Fluoxetine 40 mg daily

a Non-smoker or abstinent for least six months.

### 3.2 Pharmacokinetics

Steady-state clozapine and norclozapine pharmacokinetic parameters are shown in Table 2, Table 3. There were no statistically significant differences between Maori and European groups for any pharmacokinetic parameter, under both fed and fasted conditions. A 2-way analysis of variance indicated no significant effects of smoking, ethnicity, or their interaction on clozapine dose or pharmacokinetic parameters (data not shown).

**Table 2:** Mean (s.d.) steady state clozapine pharmacokinetic parameters by ethnic group.

	Maori (n = 12)	European (n = 16)	t^a^; p
Fasted			
AUC_0 − τ_(ng·h/mL)	3257 (845)	3056 (1311)	0.46; 0.64
C_max_ (ng/mL)	266 (55.8)	260 (81.2)	0.24; 0.81
C_min_ (ng/mL)	71.4 (23.9)	68.6 (39.1)	0.20; 0.84
t_max_ (h)	2.5^b^	2^b^	64.5^c^; 0.14

Fed			
AUC_0 − τ_(ng·h/mL)	3068(598)	2890 (1237)	0.46; 0.65
C_max_ (ng/mL)	214 (47.2)	211 (70.2)	0.11; 0.91
C_min_ (ng/mL)	69.1 (21.2)	66.5 (39.1)	0.21; 0.84
t_max_ (h)	5^b^	4^b^	61.0^c^; 0.11

a Unpaired *t*-test, df = 26.

b Median.

c Mann-Whitney *U* test.

**Table 3:** Mean (s.d.) steady state302 norclozapine pharmacokinetic parameters by ethnic group.

	Maori	European	t^a^; p
Fasted			
AUC_0 − τ_(ng·h/mL)	1736 (622)	1722 (884)	0.04; 0.97
C_max_ (ng/mL)	106 (42.4)	112 (55.6)	0.31; 0.76
C_min_ (ng/mL)	48.5 (18.3)	50.8 (29.6)	0.24; 0.81
t_max_ (h)	2^b^	2^b^	85^c^; 0.61

Fed			
AUC_0 − τ_(ng·h/mL)	1642 (523)	1640 (937)	0.01; 0.99
C_max_ (ng/mL)	92.0 (31.3)	100 (68.2)	0.40; 0.70
C_min_ (ng/mL)	47.0 (16.0)	49.3 (29.4)	0.24; 0.81
t_max_ (h)	5.5^b^	4.5^b^	77^c^; 0.39

a Unpaired *t*-test, df = 26.

b Median.

c Mann-Whitney *U* test.

### 3.3 Safety

There were no deaths or serious adverse events during the study. A total of 165 adverse events were reported by 18 subjects while taking clozapine tablets. The most common of these were drowsiness (46 reports), constipation (25 reports), dizziness (23 reports), dry mouth (16 reports), and confusion and blurred vision (15 reports each). All adverse events were of mild to moderate intensity. There were no differences in adverse event reporting between Maori and European groups, and all adverse events had resolved by study completion. There were no findings of note from safety laboratory tests, ECGs or vital signs.

## 4 Discussion

The main finding of this study is that there are no differences in steady-state clozapine or norclozapine pharmacokinetics, under both fed and fasted conditions, between Maori and NZ European patients. This finding is consistent with an earlier report of similar mean daily clozapine doses in Maori and European patients (El-Badri and Mellsop, 2011).

Based on the complexity of clozapine metabolism, it was not possible a priori to predict whether Maori and European pharmacokinetic parameters would be similar or different. Cytochrome-P450 (CYP) 1A2 is the predominant enzyme involved in clozapine metabolism, with CYP3A and CYP2C19 also involved (Jaquenoud Sirot et al., 2009). Maori have been reported to have higher frequencies of two poor metabolizer alleles for CYP2C19 (*2 and *3) compared with Caucasians (Lea et al., 2008). Because of the substantial effects of environmental factors on CYP1A2 activity, phenotyping may be preferred for assessment of activity in vivo (Faber et al., 2005). Rates of CYP1A2 poor metabolism vary by ethnic group (McGraw and Waller, 2012), but there are as yet no published data available regarding Maori.

The limitations of this study include the fact that it was based on a post-hoc analysis of participants enrolled in a bioequivalence study. Although the entire study was adequately statistically powered, analysis by ethnic subgroup was not. Post-hoc estimates indicate the study was adequately powered to detect large (30–40%) differences in pharmacokinetic parameters, and thus subtle differences might be missed. On the other hand, the between group results are remarkably similar, suggesting that the risk of a Type II error is small. A further limitation of this study is that we were unable to control for smoking status or prescription of concomitant medications, both known to potentially affect clozapine metabolism.

In conclusion, the finding of essentially identical clozapine bioavailability in Maori and European subjects suggests that this drug can be dosed in the New Zealand setting without consideration of ethnicity.

## Funding Sources

The primary clozapine bioequivalence study, on which this secondary ethnicity study was based, was a clinical trial sponsored by Douglas Pharmaceuticals Ltd. The sponsor was not involved in the data analysis, manuscript preparation, or decision to publish either report. Additional funding support for norclozapine assays was provided by the New Zealand Lottery Grants Board (application no. 353144).

## Conflicts of Interest

C-TH, FL, and NH received salaries from the contract research organisation (Zenith Technology, Dunedin, New Zealand) that completed the study. PG and CG received honoraria for advice on study design and for review of safety data. DM received payment for serving on a Data Safety Monitoring Board for a two unrelated studies also funded by Douglas Pharmaceuticals (noribogaine 2014–2015; ketamine 2016–2017).

## Author Contributions

DM conceived and planned the study of ethnicity as part of a larger bioequivalence trial organized by the authors (Glue et al., 2012). All authors also contributed conduct of the trial, drafting and approving the final manuscript. NH and PG led the laboratory and statistical procedures, respectively.
